# Supplementary material for: Bio-inspired Murray materials for mass transfer and activity
Source: Nat Commun. 2017 Apr 6;8:14921. doi: 10.1038/ncomms14921 (PMC5384213; doi:10.1038/ncomms14921)
Supplement: Supplementary Information — Supplementary Figures, Supplementary Tables and Supplementary Methods [file ncomms14921-s1.pdf]

Supplementary Figures

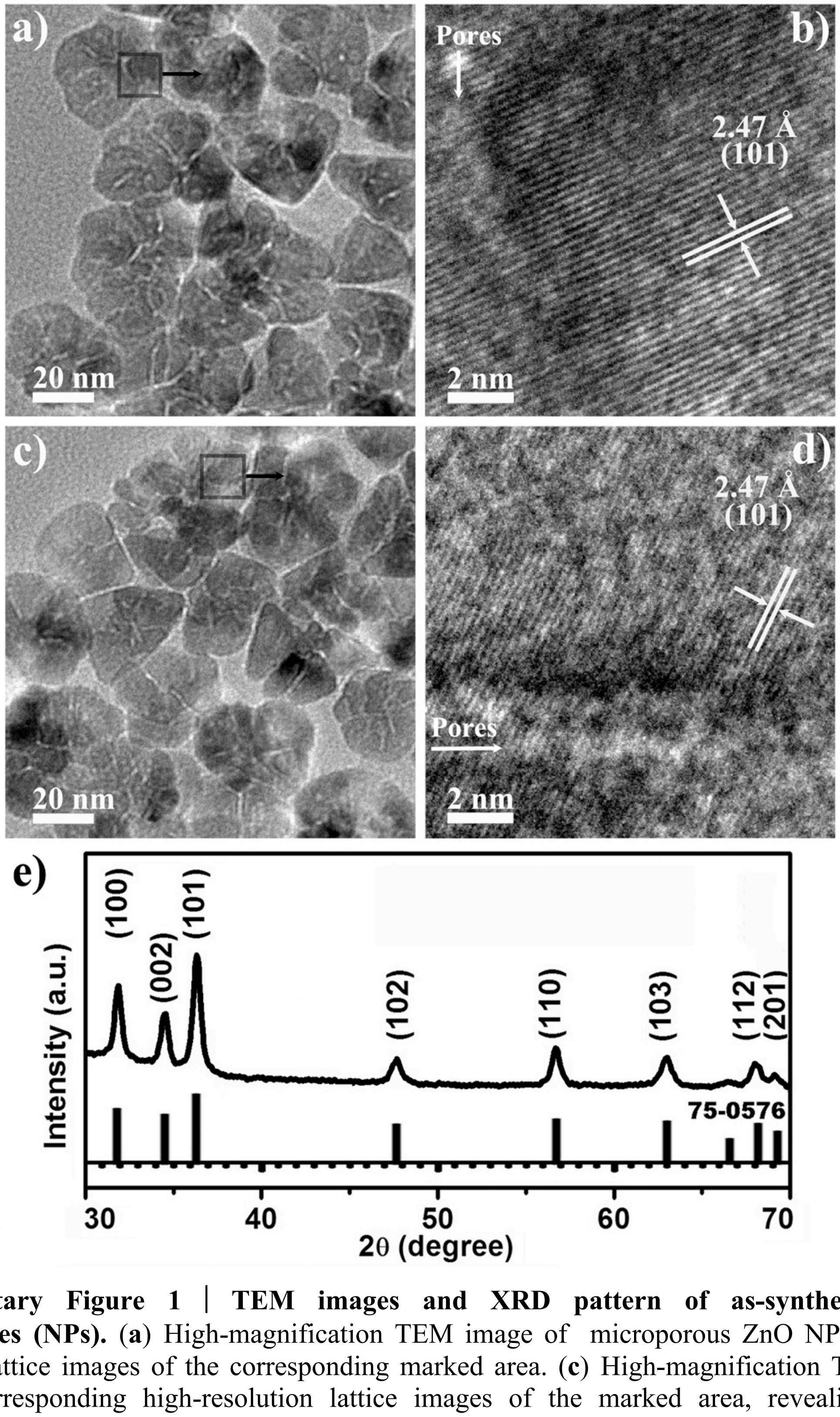

Supplementary Figure 1 | TEM images and XRD pattern of as-synthesized ZnO nanoparticles (NPs). (**a**) High-magnification TEM image of microporous ZnO NPs. (**b**) High-resolution lattice images of the corresponding marked area. (**c**) High-magnification TEM images and (**d**) corresponding high-resolution lattice images of the marked area, revealing that the microporous ZnO NPs were crystalline. (**e**) XRD pattern of as-synthesized crystallized microporous ZnO NPs, corresponding to hcp ZnO (hexagonal closed packed, JCPDS 75-0576).

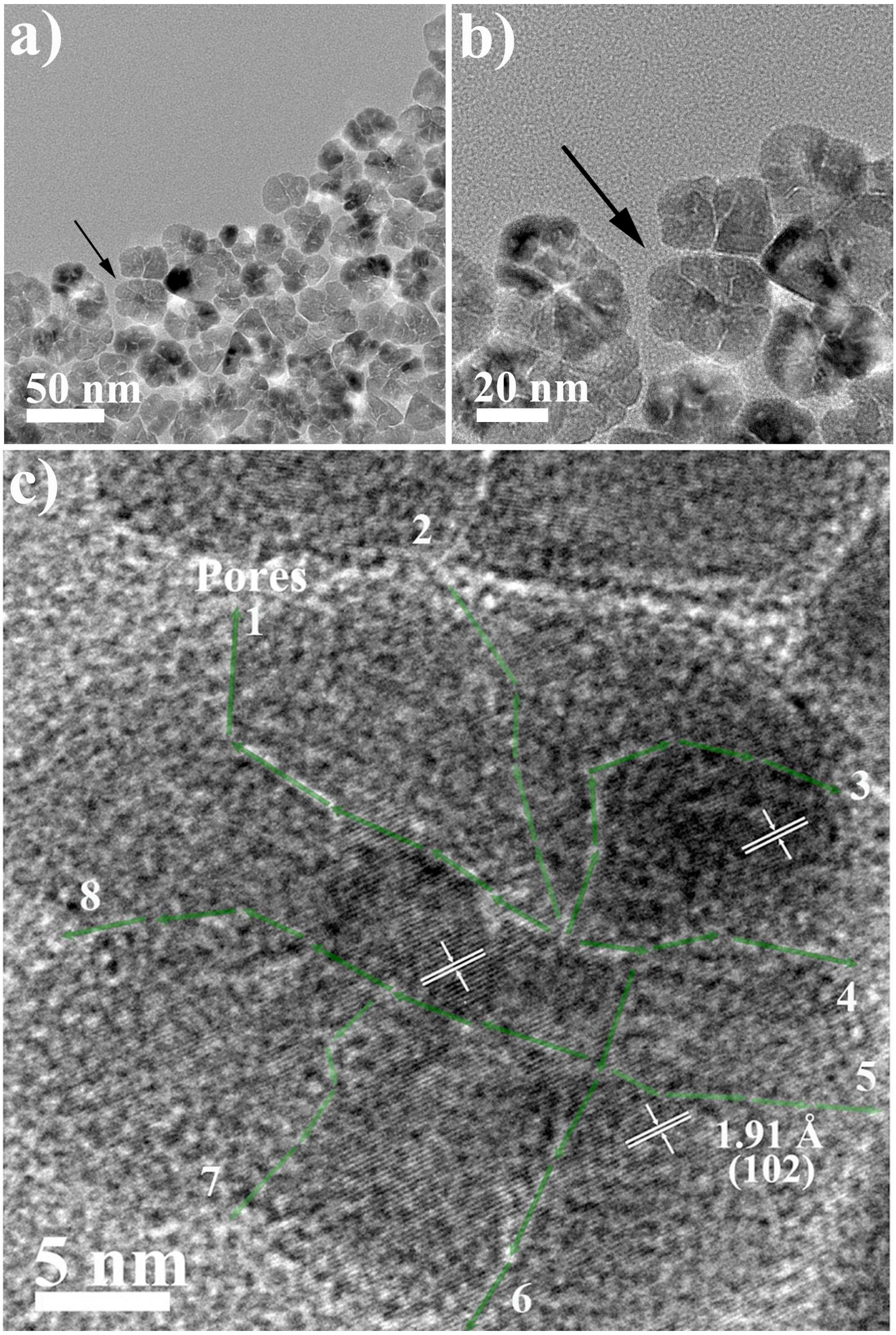

Supplementary Figure 2 | High-resolution TEM images with various magnifications of as-synthesized microporous ZnO nanoparticles (NPs). **(a, b)** TEM images of ZnO nanoparticles and (**c**) High-resolution TEM image of a ZnO nanoparticle (marked in **a** and **b**) with open microporous channels as shown by the green arrows, displaying eight counts of micropores with ~1 nm diameter in a single NP. It shows that the microporous ZnO NPs are single crystals and the crystallographic orientation of ZnO around the micropores are coincident, demonstrating a single nanocrystal with many open microprous channels passing through it.

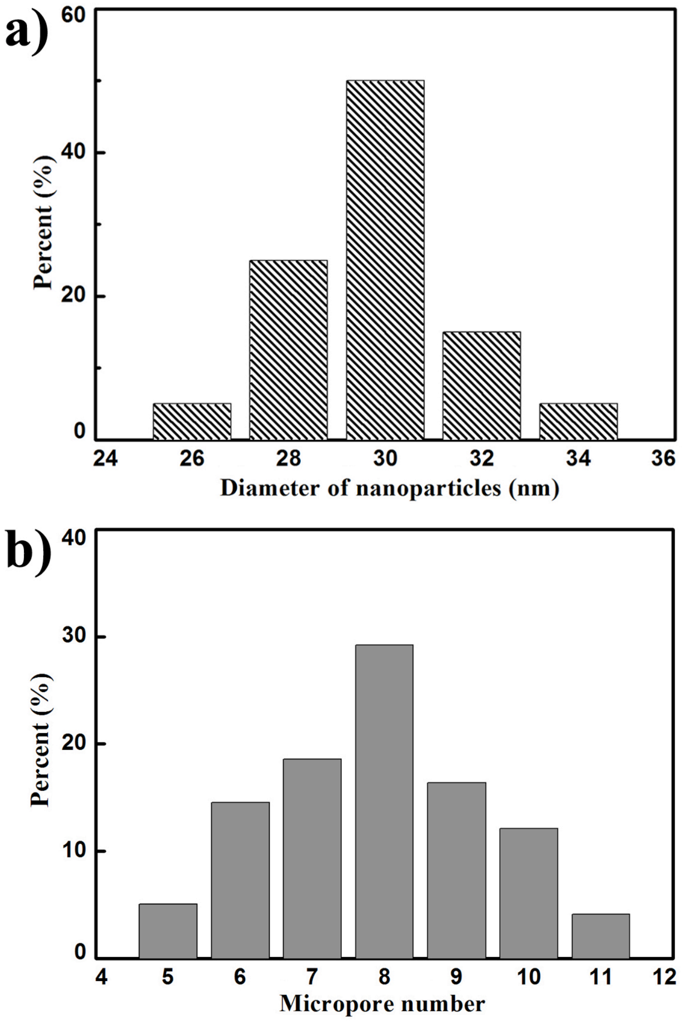

Supplementary Figure 3 | Particle-size distributions of as-synthesized microporous ZnO nanoparticles (NPs) and micropore number distributions within single NP. Histograms showing (**a**) the percentage distribution of particle-sizes and (**b**) the percentage distribution of micropore numbers within single NP obtained from counting ~300 NPs through HRTEM.

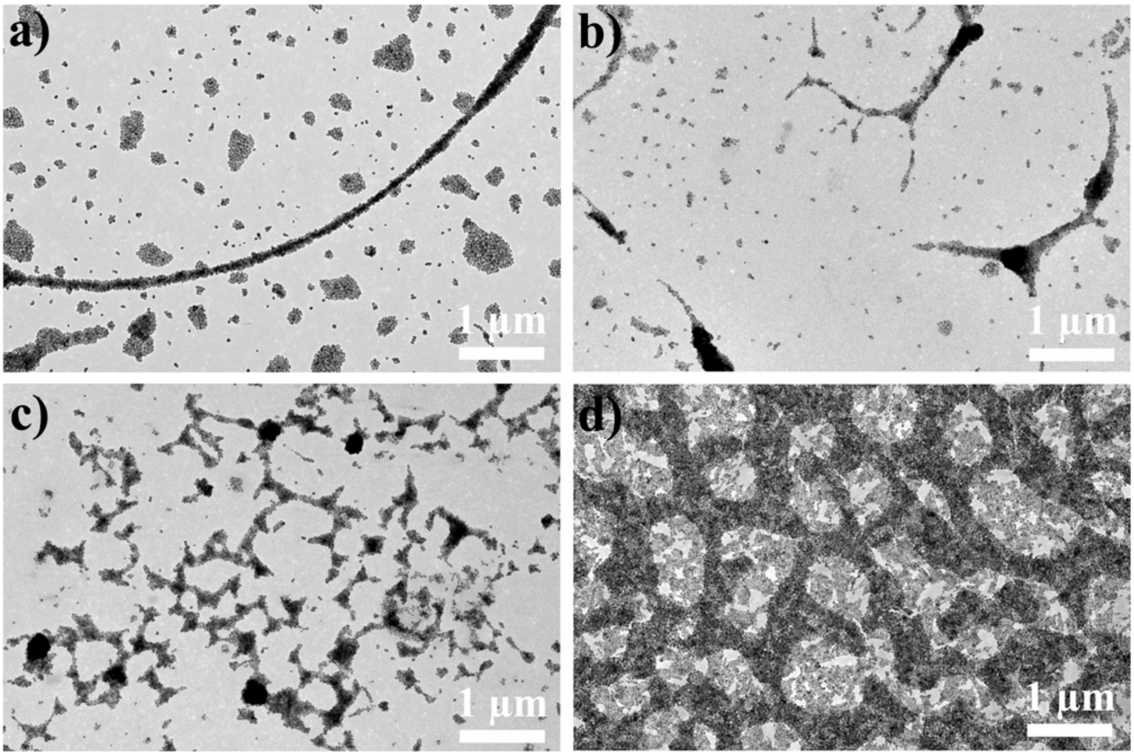

Supplementary Figure 4 | TEM images of self-assembled ZnO nanoparticles (NPs) formed via drop-casting from hexane suspensions with various nanoparticle concentrations. During the self-assembly process of the ZnO NPs under ambient conditions, hexane evaporation is directly influenced by the NPs. The nucleation density and expansion range of the vapour holes are remarkably found to be influenced by their concentration in suspension in hexane during solvent evaporation. (**a**) For 0.03125 mg/mL NP concentration, the NPs are self-assembled into isolated islands and discontinuous curves are formed from the edges of large rings, showing only a few zones of random nucleation and a favorable expansion of the holes towards larger scales. (**b, c**) Network nodes and branches, corresponding to smaller macropores, gradually emerge with increasing NP concentration to **(b)** 0.0625 and **(c)** 0.125 mg/mL. This is attributed to increased nucleation and restricted expansion of the holes. With a highly increased coverage of NPs on the substrate, very dense NPs divide the hexane into more and more droplets. These smaller nanoscale liquid pockets are more unstable and randomly evaporate, leading to increased number of vapour-induced holes. (**d**) Increasing the concentration to 0.5 mg/mL led to a porous network with superfluous NPs forming closely packed structures underneath.

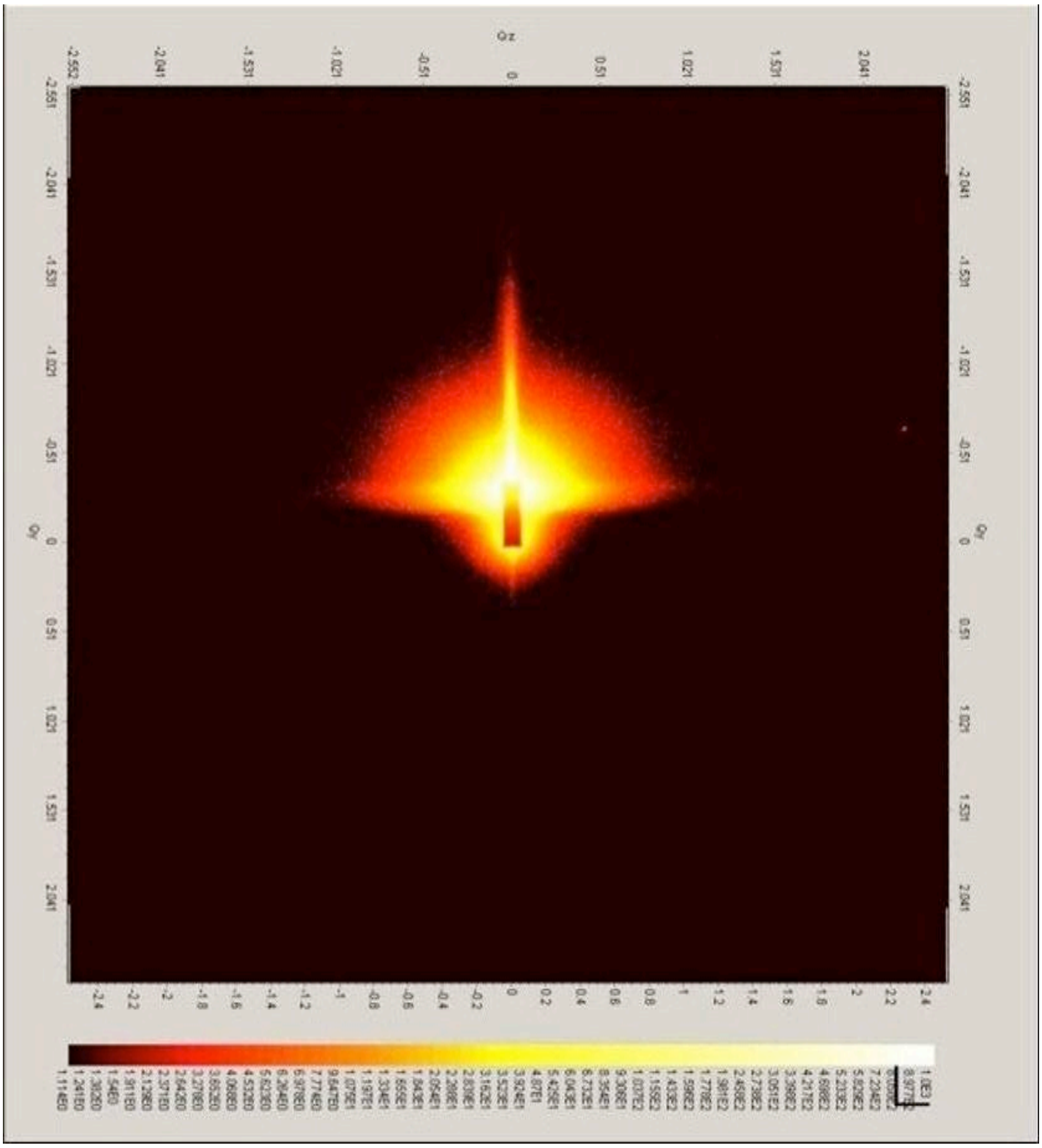

Supplementary Figure 5 | Grazing-incidence small-angle x-ray scattering data (GISAXS) acquired from micro-meso-macroporous ZnO (M-M-M) on Cu foil.

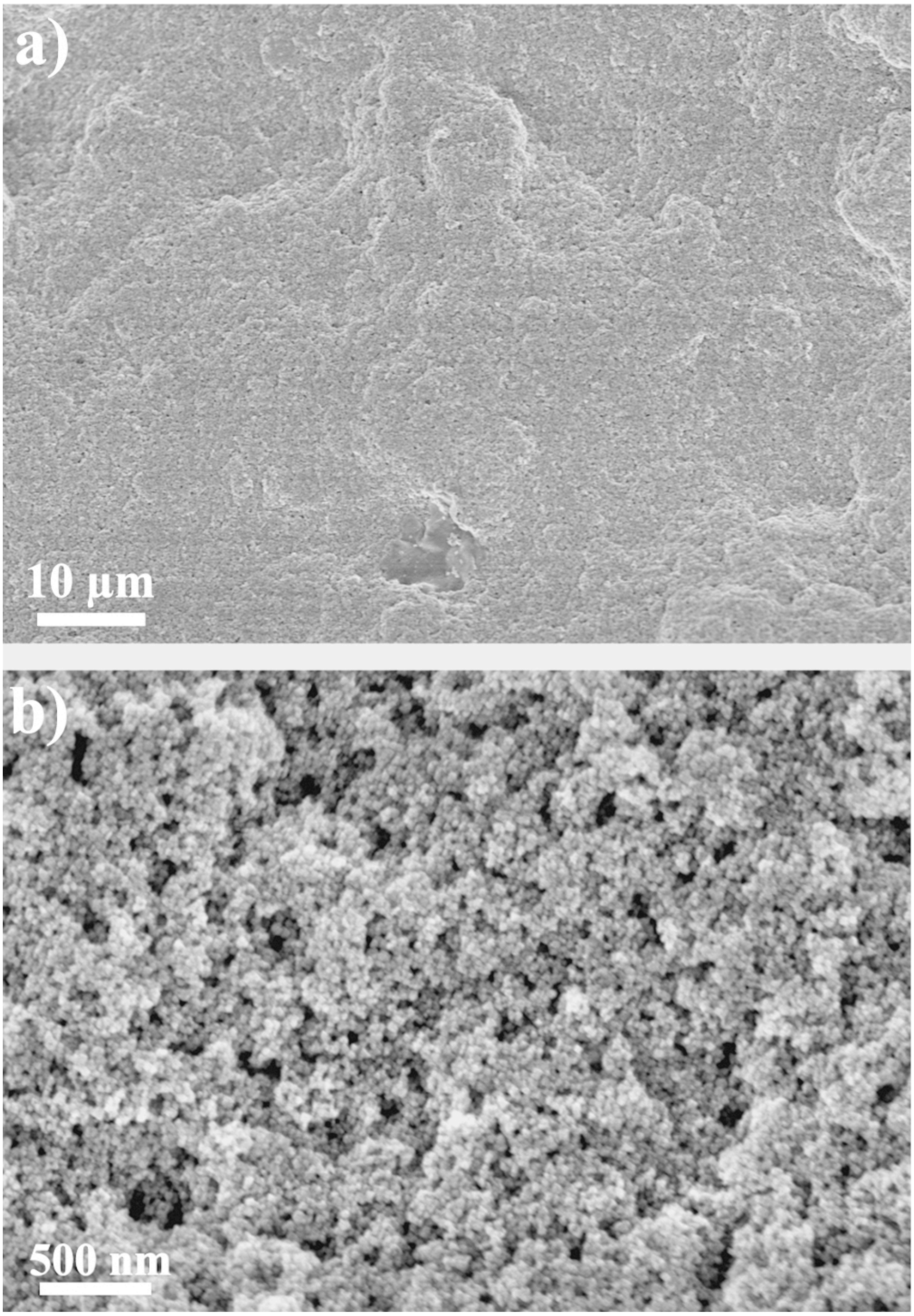

Supplementary Figure 6 | SEM images of ZnO M with solid 30 nm nanoparticles only showing interparticle mesopores. (**a**) Low-magnification and (**b**) High-magnification image. Solid 30 nm ZnO NPs are obtained by mixing $Zn(acac)_2$ (0.8 g), oleylamine (2 g), and triphenylphosphine (0.8 g) at 80 °C, and reacting them at 150 °C for 60 min and then at 290 °C for 180 min.

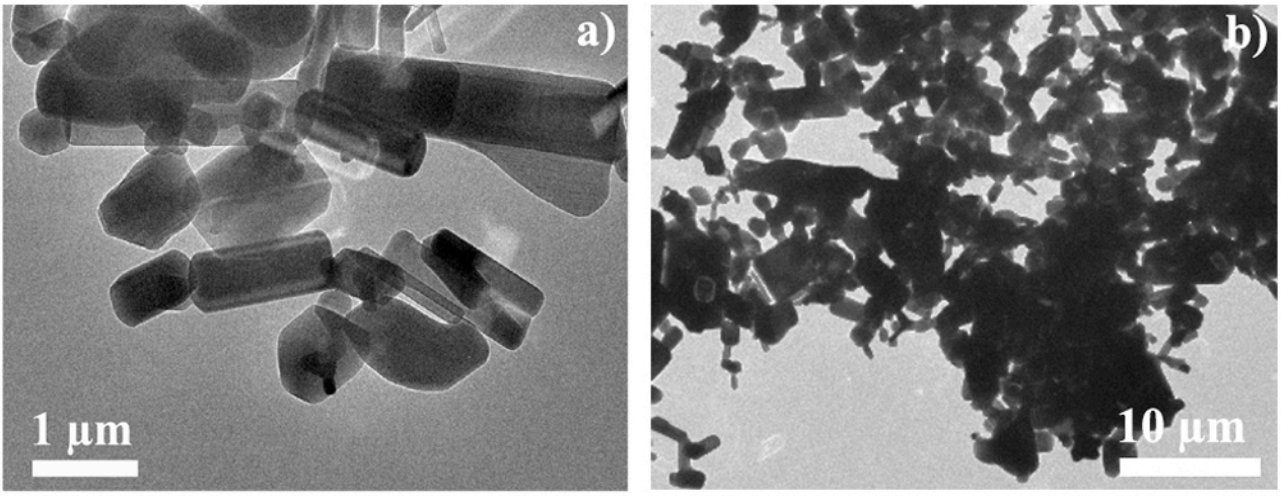

Supplementary Figure 7 | TEM images purchased ZnO bulk showing micron-sized crystals. (**a**) Low-magnification and (**b**) High-magnification image.

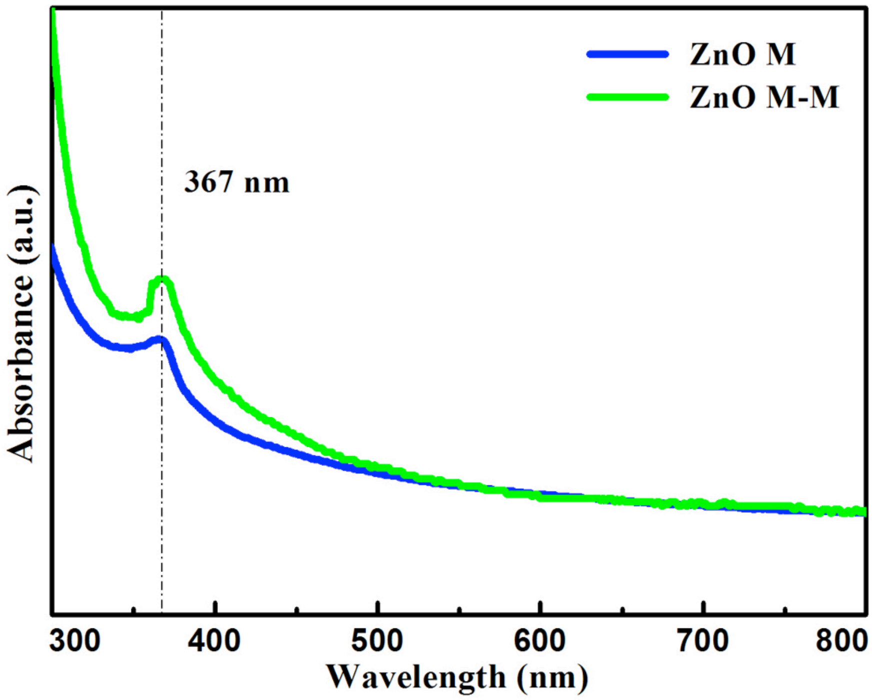

Supplementary Figure 8 | UV-vis absorption spectra of as-synthesized ZnO NPs without micropores (ZnO M) and with micropores (ZnO M-M). The optical absorption spectra were recorded from the hexane suspension of NPs using a UV-vis spectrometer (Shimadzu UV-2550).

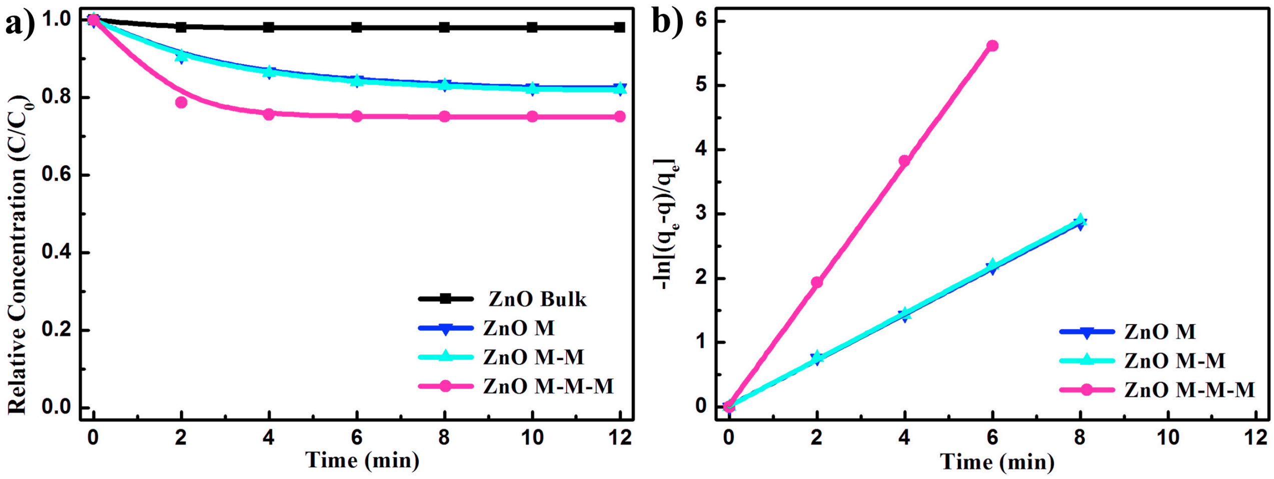

Supplementary Figure 9 | Adsorption in dark of RhB for different ZnO samples. Before the adsorption measurement, the samples are repeatedly washed using distilled water. (**a**) Variation of relative concentration of RhB (C/C_0) vs time (t) for different ZnO samples. The relative concentration is defined as the ratio C/C_0, where C (mg/l) is the residual concentration of the RhB and C_0 (mg/l) is the initial concentration of the RhB. (**b**) Pseudo-first order plots of RhB adsorption in dark on different ZnO samples as a function of adsorption time. The pseudo-first-order model for adsorption of RhB is depicted as: $\ln(q_e - q) = \ln(q_e) - kt/2.303$, where q_e (mg/g) and q (mg/g) are the adsorption capacity of RhB onto ZnO at equilibrium and at time t, respectively. And the term k (1/min) is the pseudo-first order rate constant, which can be obtained from the slope of plots of $-\ln[(q_e - q)/q_e]$ versus time t. Also, $q = (C_0 - C)V/M$, where V the solution volume (L), and M the mass of the adsorbent (mg). It reveals that the adsorption rate of ZnO M-M-M is 2.5 times higher than that of ZnO M-M or ZnO M. The residual concentration of the RhB at equilibrium is 0.75 for ZnO M-M-M, 0.82 for ZnO M-M, and 0.825 for ZnO M, respectively. Thus, we can estimate the amount of accessible Zn atoms from the adsorption measurement. The concentration of RhB aqueous solution is 10^{-5} M (10 mL) in our experiment. Based on the adsorption modes of RhB (two zinc atoms for adsorbing one RhB molecule), therefore, the amount of accessible zinc atoms for adsorbing RhB can be estimated to be 3.1×10^{-5} mol/g for ZnO M-M-M, 2.3×10^{-5} mol/g for ZnO M-M, and 2.2×10^{-5} mol/g for ZnO M, respectively.

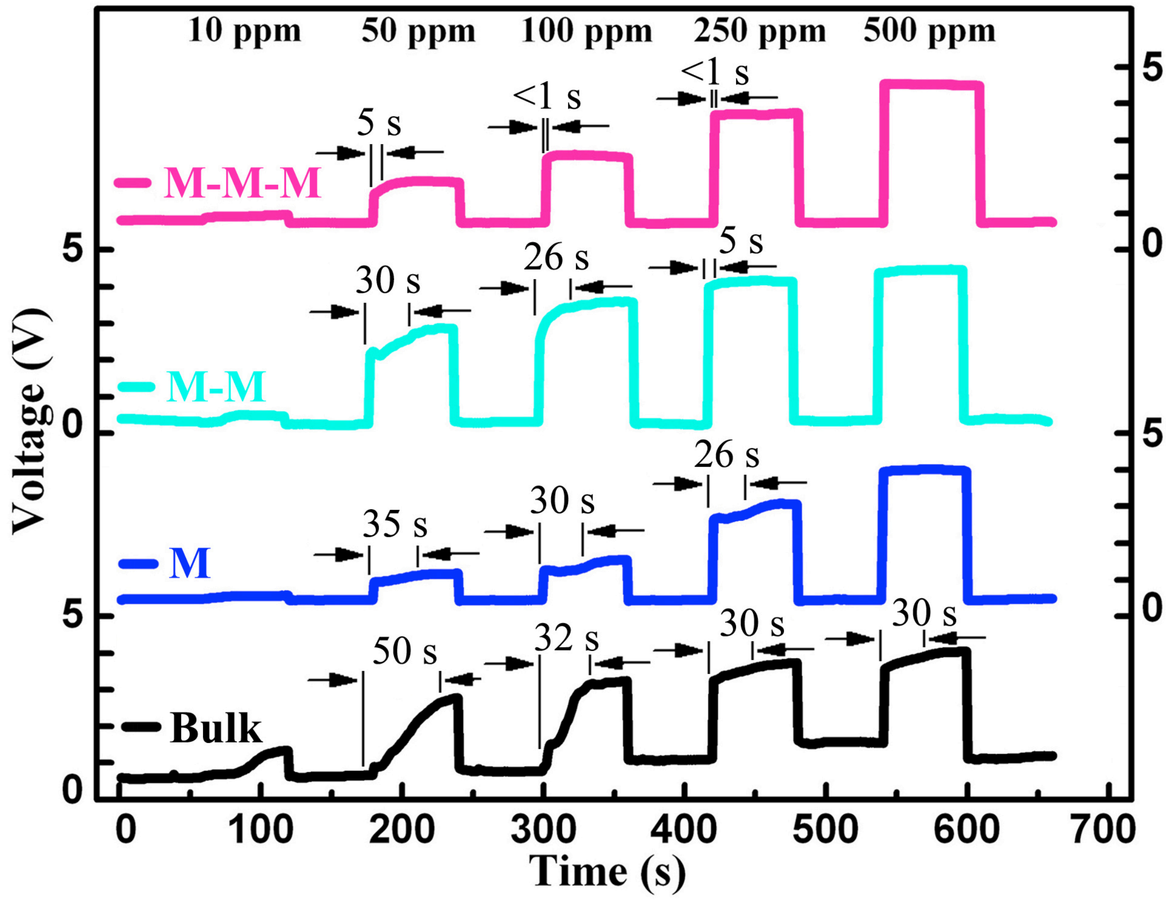

Supplementary Figure 10 | Responses of different ZnO samples exposed to ethanol vapor with various concentrations at 330 ºC. The response time to reach the 90% of the final equilibrium signal of voltage represents the response performance of gas-sensors. The response time to reach the 90% of the equilibrium value for ZnO Bulk is very slow from 50 to 500 ppm. ZnO M exhibits a response time > 25 s at 50, 100 and 250 ppm. ZnO M-M exhibits a response time > 25 s at 50, and 100 ppm. The response for ZnO M-M-M is very fast, within 5 s at 50 ppm, < 1 s at 100-500 ppm. The ZnO M-M-M demonstrated much faster response than that of others, especially for gas-sensing ethanol vapor with low concentrations.

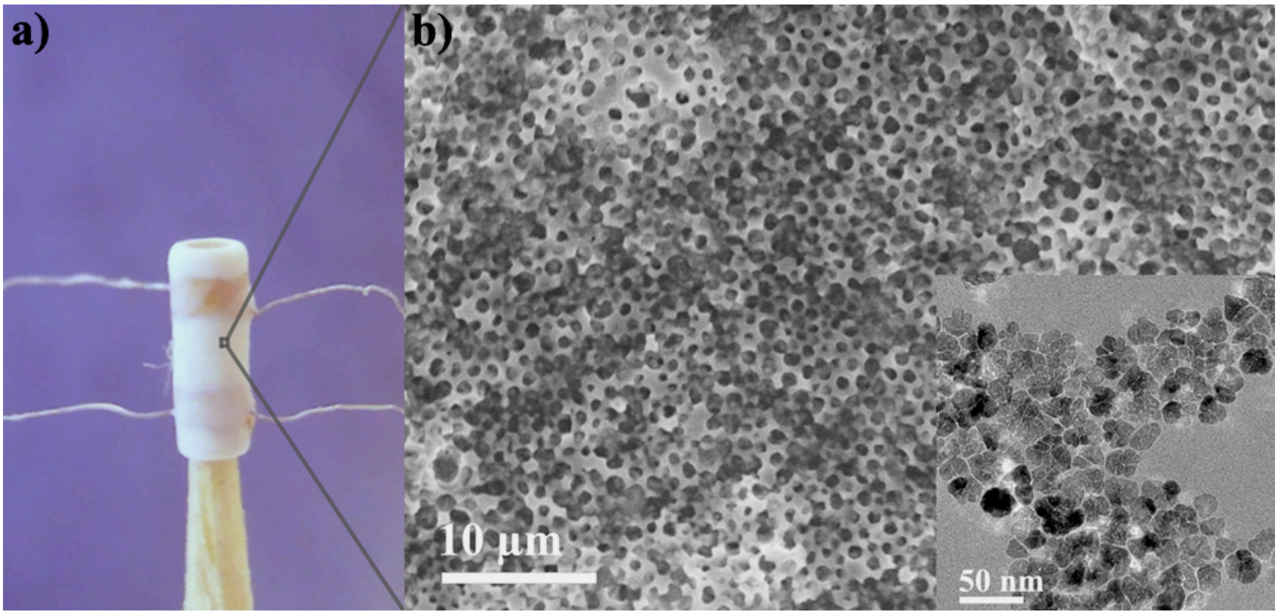

Supplementary Figure 11 | Images for gas-sensing using ZnO M-M-M. (**a**) Photograph of the sensor setup. (**b**) SEM and (inset) TEM image of ZnO M-M-M with macro-meso-micropores from the ceramic tube shown in (a) after repeated gas-sensing measurement.

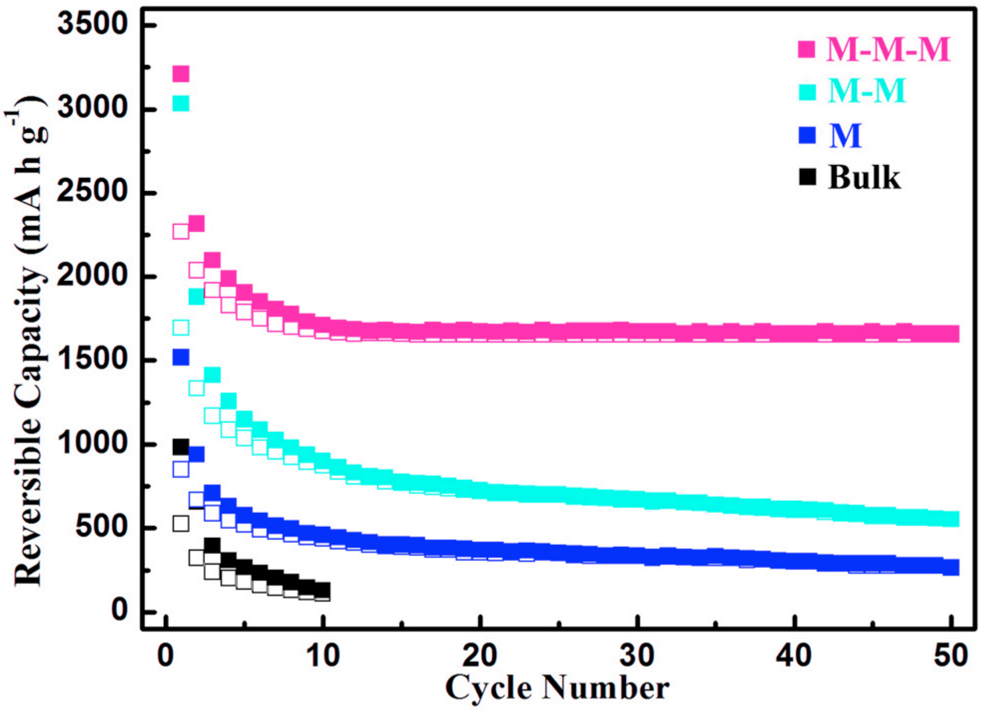

Supplementary Figure 12 | Comparison of cycling performance for different ZnO samples at a current density of 0.05 A g^{-1}.

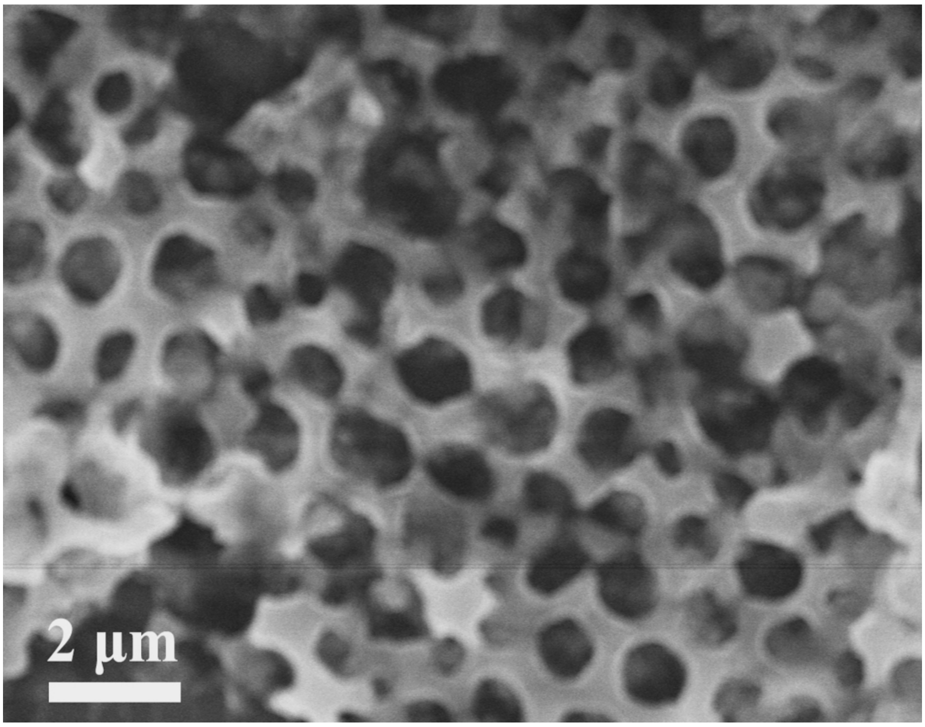

Supplementary Figure 13 | SEM images of the ZnO M-M-M electrode after 5000 cycles at 2.5 A g^{-1} for Li-storage.

Supplementary Tables

Sample	Macropore size [nm]	Mesopore size [nm]	Micropore size [nm]	S $[m^2\ g^{-1}]$	S_{micro} $[m^2\ g^{-1}]$	Particle size [nm]
ZnO M-M-M	1000	18	1.1	76	38	30
ZnO M-M	−	19	1.1	76	38	30
ZnO M	−	22	−	32	−	30
Bulk ZnO	−	−	−	3	−	>1000

Supplementary Table 1 | Pore size, BET surface area and average particle size of the ZnO samples. Mercury intrusion porosimetry is used to obtain the macropore distribution. Analysis of argon adsorption-desorption isotherms is used to obtain mesopore distribution, micropore size, and the BET surface-area of NPs and the surface-area of micropores. The average particle sizes are obtained from TEM observations. To fabricate 3D network films, microporous ZnO NPs are suspended in hexane (ZnO M-M-M) and in ethanol (ZnO M-M) at ~0.25 mg/mL concentration and repeatedly drop-cast on to 20 mm × 20 mm Si wafers. Similarly, solid 30 nm ZnO NPs (ZnO M) and ZnO micron-powder (bulk sample) are separately dispersed in ethanol at ~0.25 mg/mL concentration and repeatedly drop-cast on to 20 mm ×20 mm Si wafers.

Sample	Gas sensitivity (R_{air}/R_{gas})			Response time (s)		
	100 ppm	250 ppm	500 ppm	50 ppm	100 ppm	250 ppm
ZnO M-M-M	93	419	457	5	< 1	< 1
ZnO M-M	40	93	105	30	26	5
ZnO M	21	51	57	35	30	26
Bulk ZnO	9	11	11	50	32	30

Supplementary Table 2 | Gas sensitivity and response speed of ZnO samples. The sensitivity is defined as the ratio R_{air}/R_{gas}, where R_{air} is the electrical resistance measured in air and R_{gas} is that measured in the test gas atmosphere. The response time to reach the 90% of the final equilibrium signal of voltage represents the response speed of gas-sensors.

Supplementary Methods

Derivation of Murray's law for mass transfer involving no mass variations. Murray's law is based on optimizing mass transfer by minimizing transport resistance in pores with a given volume. In the paper published in PNAS in 1926, based on consideration of how to ensure nutrition transfer with full coverage and fluency as a precondition, Murray deduced that the cost of operation of physiological systems tends to be a minimum for optimum substance transfer networks and formulated what is now known as Murray's law. Murray derived his law for optimal cardiovascular design that defines the sizes of blood vessels from the aorta through progressive branch points to the capillaries. Like the laws of Poiseuille and Fick, which were also formulated from a biological context, Murray's law is a basic physical principle for transfer networks.

Sherman and Shan et al. have theoretically derived Murray's law for optimizing mass transfer involving no mass variations. The simplified derivation process is presented below. For an individual circular pore with radius of r and length of l, the volume (V) of the pore is $\pi r^2 l$. For laminar flow, the Hagen-Poiseuille's law gives: $Q_1 = \frac{\pi r^4}{8\mu}\frac{\Delta P}{l}$, where Q_1 is the volumetric flow rate, μ is the viscosity and ΔP is the pressure drop across the length of the pore. According to Murray's principle, the optimization function can be written by introducing a Lagrange multiplier λ to minimize the entransy dissipation rate:

$$\frac{\partial[Q_1\Delta P - \lambda V]}{\partial r} = 0 => \frac{\partial[\frac{\pi r^4}{8\mu}\frac{\Delta P^2}{l} - \lambda\pi r^2 l]}{\partial r} = 0 \Rightarrow \frac{\Delta P}{l} = \frac{1}{r}\sqrt{4\mu\lambda}\,.$$

Thus, $Q_1 = \frac{\pi}{\sqrt{16\,\mu/\lambda}} r^3 \Rightarrow Q_1 \propto r^3 \Rightarrow Q_1 = k_1 r^3$, where k_1 is a constant.

Murray's principle can be easily extended to other transfer phenomena. Based on Murray's principle, Sherman and Shan et al. have also theoretically derived and developed similar formulae for mass diffusion in a circular pore filled with gas or liquid, and for ionic or electronic transfer in a

circular pore filled with electrolytic solution. The simplified derivation process is presented as follows.

For mass diffusion, Fick's law gives, $Q_2 = D\pi r^2 \frac{\Delta C}{l}$, where Q_2 is the amount of substance diffused through a given cross-section per unit time, D is the diffusion coefficient and ΔC is the concentration difference across the pore. Again, the optimization function can be written by introducing a Lagrange multiplier λ to minimize the entransy dissipation rate, according to Murray's principle: $\frac{\partial[Q_2 \Delta C - \lambda V]}{\partial r} = 0$ => $\frac{\partial[D\pi r^2 \frac{\Delta C^2}{l} - \lambda \pi r^2 l]}{\partial r} = 0 \Rightarrow \frac{\Delta C}{l} = \sqrt{\frac{\lambda}{D}}$.

Thus, $Q_2 = \pi\sqrt{\lambda D} r^2 \Rightarrow Q_2 \propto r^2 \Rightarrow Q_2 = k_2 r^2$, where k_2 is a constant.

For ionic or electronic transfer, Ohm's law and Pouillet's law give, $Q_3 = \sigma\pi r^2 \frac{\Delta V}{l}$, where Q_3 is the electric charge transferred through a given cross-section per unit time, σ is the conductivity and ΔV is the potential difference across the length of the pore. Similarly, $\frac{\partial[Q_3 \Delta V - \lambda V]}{\partial r} = 0$ => $\frac{\partial[\sigma\pi r^2 \frac{\Delta V^2}{l} - \lambda \pi r^2 l]}{\partial r} = 0 \Rightarrow \frac{\Delta V}{l} = \sqrt{\frac{\lambda}{\sigma}}$.

Thus, $Q_3 = \pi\sqrt{\lambda\sigma} r^2 \Rightarrow Q_3 \propto r^2 \Rightarrow Q_3 = k_3 r^2$, where k_3 is a constant.

Therefore, for optimizing mass transfer according to the Murray's principle: $Q = kr^\alpha$, where the exponent α (2 or 3) is dependent on the type of the transfer. For connecting a parent pipe with radius of r_0 to many children pipes with radius of r_i for mass transfer with no mass variations, the law of mass conservation at a junction gives: $Q_0 = \sum_{i=1}^{N} Q_i$. Therefore, Murray's law for optimizing mass transfer involving no mass variations can be written as $r_0^\alpha = \sum_{i=1}^{N} r_i^\alpha$. For laminar flow transfer, α =3; For mass diffusion or ionic transfer, α =2.

It can be seen that by optimizing the transfer in all the pores connected with a given volume to ensure mass transfer with full coverage and fluency, the networks should support a volume-preserving branching for Laminar flow or an area-preserving branching for ionic or electronic transfer. By evolution and natural selection, living organisms have firstly shaped their porous structures based on Murray's law. In plant stems and leaf veins, the sum of radii cubed of pores remains constant across every branching, to enhance flow conductance which is proportional to photosynthesis. For insect larvae relying upon gas diffusion for breathing, the sum of radii squared of their tracheal pores remains constant along the transfer pathway to improve gas delivery of CO_2 and O_2 by about 10^4 and 10^6 times faster in air than in water or tissues.
